# Characterization of *Leishmania* spp. Causing Cutaneous Lesions with a Negative Parasitological Diagnosis in Panama

**DOI:** 10.3390/tropicalmed7100282

**Published:** 2022-10-03

**Authors:** Adelys M. Reina, Juan Castillo Mewa, José E. Calzada, Azael Saldaña

**Affiliations:** 1 Departamento de Investigación en Parasitología, Instituto Conmemorativo Gorgas de Estudios de la Salud, Panama 0816, Panama; 2 Departamento de Investigación en Genómica y Proteómica, Instituto Conmemorativo Gorgas de Estudios de la Salud, Panama 0816, Panama; 3 Centro de Investigación y Diagnóstico de Enfermedades Parasitarias (CIDEP), Facultad de Medicina, Universidad de Panamá, Panama 0824, Panama

**Keywords:** *Leishmania (Viannia) panamensis*, diagnosis, cutaneous leishmaniasis, parasite load, molecular characterization, Panamá

## Abstract

A total of 123 DNA samples from Panamanian patients with cutaneous leishmaniasis (CL) lesions were evaluated. These samples were previously confirmed with CL by a specific KDNA-Viannia PCR but had a negative parasitological diagnosis (Group A). Epidemiological variables, such as age, sex, geographic origin, evolution time, and the number and location of the lesions, were analyzed. No significant differences (*p* < 0.05) were found when these variables were evaluated against a control panel of 123 CL lesion samples from CL patients with positive parasitological diagnoses (Group B). Of the 123 samples (Group A), 67% (82/123) gave positive results when re-analyzed by PCR-hsp70. An analysis of 69 of these samples via PCR-hsp70-RFLP showed that 59.4% (41/69) of the found restriction patterns corresponded to *Leishmania (Viannia) panamensis* and 40.6% (28/69) to *Leishmania (Viannia) guyanensis*. Finally, the sequence and phylogenetic analysis of 32 of the samples confirmed the species in 21 (65.6%, 21/32) samples, originally characterized as *L. (V.)* panamensis. However, 11 samples (34.4%, 11/32), initially identified via RFLP-Hsp70 as *L. (V.) guyanensis*, matched the sequence of a genetic variant known as *Leishmania* sp.1. These results point out the species/genetic variants of *Leishmania* in the case of CL lesions with an apparently low parasite load.

## 1. Introduction

Cutaneous leishmaniasis (CL) is a parasitic disease caused by hemoflagellates of the genus *Leishmania*, which are transmitted by small dipterans of the subfamily phlebotominae [1,2]. Human infections with CL are characterized by chronic lesions that compromise the skin and/or mucous membranes [3]. The parasitological diagnosis of this infection is based on the microscopic finding of the parasite in stained smears and via isolation of the parasite in cultures from samples obtained from skin lesions [4,5]. Direct microscopy for CL diagnosis has high specificity but has sensitivities that vary between 40 and 74.4% [6,7,8]. Microscopic visualizations of *Leishmania* amastigotes in stained smears depend on various factors, including *Leishmania* species, parasitic load, evolution time of the lesion, added bacterial contamination, and the technical execution of the analysis [9,10,11]. The implementation of molecular techniques has significantly improved the relatively low sensitivity exhibited by microscopic methods, particularly when used for the diagnosis of CL lesions with a low parasite load [10,12,13,14,15]. 

*Leishmania (V.) panamensis* has been the main etiologic agent of CL reported in Panamá since it was accepted as a new species in 1972 [16]. However, recent studies have confirmed that at least one genetic variant of *L. (V.) panamensis* is also frequently found in Panamanian patients with CL [17,18]. These studies have also confirmed the presence of other species circulating in the country, such as *L. (V.) braziliensis*, *L. (V.) guyanensis*, and *L. (V.) naiffi* at low frequencies. [17,18]. However, there is always the possibility of greater genetic diversity in *Leishmania* parasites infecting humans in the country. This is reinforced by the description of other genetic variants of *L. (V.) panamensis* in geographic regions close to Panamá [19,20].

This study was conducted to determine (by molecular analysis) the *Leishmania* species present in the lesions of Panamanian patients who were diagnosed with CL via PCR but that had a negative parasitological preliminary diagnosis (smear and culture). Our findings confirmed that *L. (V.) panamensis* is the predominant species in these lesions with an apparently low parasite load. However, the presence of a genetic variant within this species was also frequently found.

## 2. Materials and Methods

### 2.1. Study Design and DNA Samples

A descriptive and analytical study was performed, where DNA samples (isolated from skin lesions of patients who attended the Unidad de Diagnóstico, Investigación Clínica y Medicina Tropical at ICGES, Panamá, between 2015 and 2019) were retrospectively evaluated. Two different groups were evaluated. One group (Group A) consisted of 123 DNA samples from the lesions of CL patients confirmed by PCR but with a negative parasitological (smear and culture with skin scrapings) initial diagnosis. The second group comprised a panel of 123 matched DNA samples from CL lesions with a positive parasitological and molecular diagnosis (Group B). All DNA samples were maintained at the Biobank from the Departamento de Investigación en Parasitología (DIP, ICGES) and were previously analyzed by smear and/or culture and kDNA-PCR [21] as part of routine diagnosis performed at DIP-ICGES as the national reference center for CL diagnosis in the country. The use of the clinical samples and data was reviewed and approved by the National Review Board (Comité Nacional de Bioética en Investigación, Instituto Conmemorativo Gorgas de Estudios de la Salud, Panama City, Panama, 733/CBI/ICGES/19).

### 2.2. Molecular Diagnosis and Characterization

- PCR-hsp70 analysis

DNA samples from both groups were analyzed via PCR-hsp70 using primers F25 and R1310 which amplify a 1286 bp product from the heat shock protein 70, a nuclear gene specific to the *Leishmania* genus. Amplification reactions were performed in a final volume of 50 μL containing 25 μL of GoTaq Green Master Mix 2X (Promega, Madison, WI, USA), 2 μL of each primer with a final concentration of 0.6 μmol/L, F25 (5’ GGACGCCGGCACGATTKCT 3’) and R1310 (5’ CCTGGTTGTTGTTCAGCCACTC-3’), 16 μL of quality water molecular biology, and 5 μL of sample DNA or reference isolates: *L. (V.) panamensis* (MHOM/PA/71/LS-94), *L. (V.) guyanensis* (MHOM/BR/75/M4147) and *L. (V.) braziliensis* (MHOM/BR/75/M2903) [22,23,24]. The thermocycler MiniAmp™ (Applied Biosystems, Waltham, MA, USA) was used and the amplification was performed according to the following cycle: 94 °C for 5 min, 33 cycles of 94 °C for 30 s, 61 °C for 1 min, and 72 °C for 3 min; then, 72 °C for 10 min and indefinitely at 4 °C.

- PCR-hsp70-RFLP analysis

To determine the species of *Leishmania* involved in the infection, the hsp70-PCR products (1286pb) were digested with Hae III endonucleases. If the pattern was similar to the patterns of the species of the subgenus *Viannia*, they were subsequently digested with BccI (this enzyme distinguishes *L. (V.) panamensis* from *L. (V.) guyanensis*). Digestions were performed with 0.2 µL of Enzyme Hae III, with a final concentration of 2 U/ µL; 2 µL buffer (NE Buffer 2) with a final concentration of 1X, 2–15 µL of PCR product; 20 µL was completed with quality molecular water. Regarding the BccI enzyme: 0.2 µL of Enzyme with a final concentration of 2 U/µL; 0.2 µL of BSA with a final concentration of 1X were used, 2 µL of Cut Smart Buffer with a final concentration of 1X, 2–15 µL of the PCR product, completed 20 µL with molecular grade water. Reactions were incubated overnight at 37 °C and fully analyzed by electrophoresis on a 3% agarose gel for small fragments and band patterns compared with described reference species [22,23,24,25,26].

- Hsp70 sequencing and phylogenetic analysis

Additionally, the hsp70-PCR products were electrophoresed on 1.5% agarose gels in 1XTBE (89 mM T-trisborate, 2 mMEDTA, pH 8.3). The respective bands were cut from the agarose gel and the DNA was purified with the Qiaquick gel extraction kit (Qiagen^TM^, Hilden, Germany) following the manufacturer’s instructions. DNA sequencing was performed using the BigDye Terminator 3.1 sequencing kit (Applied Biosystems^TM^, Foster, CA, USA) and primers F25, R1310, 6F (5’ GTGCACGACGTGGTGCTGGTG 3’), and R617 (5’ CGAAGAAGTCCGATACGAGGGA 3’). The clean sequencing reaction clean-up was carried out using the BigDye Xterminator purification kit (Applied Biosystems^TM^, Waltham, MA, USA) [17]. The capillary electrophoresis was done in an ABI 3500xL sequencer. Chromatograms were subsequently evaluated and edited with the Sequencher 4.1.4 assembly tool (v. 4.1.4; Gene Codes Corporation, Ann Arbor, MI, USA). Then, the sequences were aligned in multiple using the MEGA X software. The nucleotide sequence distances were estimated with the Kimura-3 parameter model and 500 bootstrap replicates to build the phylogenetic tree by the Maximun Likelihood method using the package of MEGA X software. Nucleotide sequences from this study were submitted to the GenBank™ database.

### 2.3. Data Analysis 

The basic clinical-epidemiological information from each evaluated patient was obtained from the database, which is maintained at the DIP-ICGES. The following variables were studied: age, sex, geographic origin, lesion size, number and body location of the lesion, evolution time, and parasitological and molecular diagnostic results. However, for some patients, the complete referred data were not available (Table 1). The z test and Chi-square were used to compare the corresponding variables from groups A and B. Data were analyzed using JMP (SAS institute) software version 14 (JMP^®^, Version 14. SAS Institute Inc., Cary, NC, USA). Significant differences between the groups were considered when *p* < 0.05. 

## 3. Results

### 3.1. Clinical and Epidemiological Data 

In both groups, males were more frequently affected by CL than females (*p* < 0.001), and most cases corresponded to the age group of 21–50 years (Table S1). Most of the patients included in this study (in both groups) came from the central provinces of Panamá 107/246 (43%) and Panamá Oeste 75/246 (30%), followed by the province of Colón 41/246 (17%) (Table S1). After revising the travel history of each patient, these three provinces were also the probable origin of most of the infections. In group A, most of the lesions were in the legs 50/123 (41%) and face 14/123 (11%), while in group B, most of the lesions were in the arms 69/123 (56%). In both groups, around 195/246 (79%) of the patients presented a single visible lesion. Those body regions with more than two lesions were the arms in group A, 9/43 (21%), and the legs in group B, 11/30 (36%) (Table 1). No significant differences were observed when comparing the frequencies of demographic, epidemiological, and clinical variables analyzed in this study between both groups (Table 1). 

### 3.2. Leishmania Molecular Diagnosis and Characterization

Of the 123 samples that had a negative parasitological diagnosis (Group A), 67% (82/123) were positive when analyzed by PCR-hsp70, whereas all 123 samples with a positive parasitological diagnosis (Group B) had a positive result from this PCR. It was possible to type (via hsp70-RFLP at the species level) 69 (84.1%) of the 82 samples from group A with a positive result when using PCR. Of these, 41(59.4%) presented an electrophoretic digestion profile that corresponded to *L. (V.) panamensis*, and 28 (40.6%) to *L. (V.) guyanensis*. In group B, 99 samples (80.5%) were identified as *L. (V.) panamensis*, and 24 (19.5%) as *L. (V.) guyanensis* (Table S2).

### 3.3. Leishmania hsp70-Sequencing and Phylogenetic Analysis

**Figure 1:**
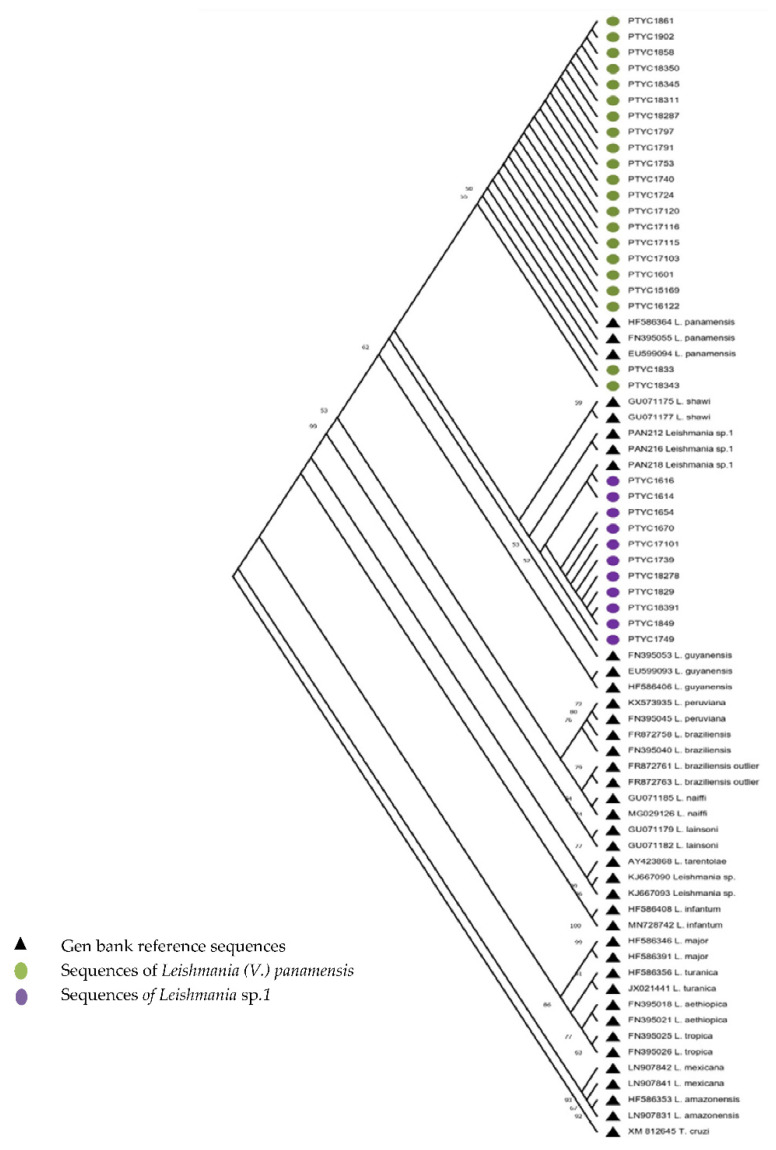
Maximum Likelihood Phylogenetic Tree based on the alignment of 1286 nucleotides from the hsp-70 gene sequence. Distances were estimated using the Kimura-3 parameter model. Reference sequences (gen hsp70) appear with a black triangle. The sequences of group A: *Leishmania (V.) panamensis* (*n* = 21) and *Leishmania* sp.1 (*n* = 11) are identified with green and purple circles, respectively. The sequences of group B, being similar, were not included.

Typing results initially obtained by PCR-hsp70-RFLP were confirmed by sequencing 32 hsp70-PCR products from group A. The remaining 37 samples from this group could not be sequenced, probably due to the low amount and quality of the DNA. A total of 21 (21/32, 65.6%) of these sequences, initially characterized as *L. (V.) panamensis* by PCR- hsp70-RFLP, presented sequences similar to the references of the *L. (V.) panamensis* sequence (Figure 1). However, the rest of sequences (11/32, 34.4%), which were initially typed as *L. (V.) guyanensis*, were clustered into a distinct group, previously named as *Leishmania* sp.1 [17]. This distinct group is positioned close to the reference sequences of *L. (V.) panamensis*, *L. (V.) guyanensis*, and *Leishmania (Viannia) shawi*, and it was conceived as a *L. panamensis* variant (Figure 1). In group B, the sequences of 99 samples (99/123, 80.5%) characterized by hsp70-RFLP as *L. (V.) panamensis* corresponded with the reference sequences of this species. However, similar to the discrepancy observed with group A, 24 sequences, initially typed as *L. (V.) guyanensis* (24/123, 19.5%), were clustered with the *L. (V.) panamensis* variant, referred to as *Leishmania* sp.1.

## 4. Discussion

CL is an important public health problem in developing countries, including Panamá. In this study, the epidemiological characteristics of patients with a negative parasitological diagnosis (group A) were evaluated against group B, who had a positive parasitological diagnosis; the results obtained agree with what is generally reported for LC cases in Panama [27]. Several methods and tools have been developed over recent years for the detection, quantification, and identification of the parasite of the genus *Leishmania* [28], but the diagnosis continues to be an objective of attention by the scientific community, who are interested in the availability of sensitive and specific methods that allow identification to occur in a timely manner to achieve effective treatment, adequate follow-ups, and take the necessary measures for the patient, with these interests being stimuli for the development of techniques based on molecular detection [29].

Although microscopic examination for the detection of *Leishmania* parasites in stained smears has shown moderate sensitivity (~70%), this methodology has proven to be practical, relatively rapid, and inexpensive for the diagnosis of most CL cases [6,7,8,13,30]. However, parasitological tests are not always conclusive for CL diagnosis [31]. For example, in endemic areas, parasitological diagnosis of LC often shows lower sensitivity, in part because, in this neglected setting, the clinical laboratories often lack the proper equipment, sampling, and processing methods. In addition, the experience of the field microscopist is limited, and the diagnosis is generally based on a single test [13,31,32]. In addition, it is also necessary to consider that the evolution of CL lesions is often accompanied by a decrease in the parasitic load, complicating parasitological diagnosis in many chronic lesions [32]. In general, molecular methods are an excellent alternative for the early detection of patients infected with *Leishmania* parasites, which helps to rapidly establish timely treatment, as well as characterize the parasite species involved [33]. Based on this observation, numerous studies argue that molecular methods represent a reliable, specific, and sensitive alternative that should be part of the routine diagnostic algorithm for CL in the endemic regions of Latin America [4,12,32]. In this study, *Leishmania* parasites present in the lesions of Panamanian patients with negative parasitological results (smear/culture) were characterized. Of the 123 evaluated samples, only 67% were positive when analyzed by PCR-hsp70, which might be explained by the low sensitivity of this test. The following analyses by PCR hsp70-RFLP showed that, from the 69 samples evaluated, 41 corresponded to *L. (V.) panamensis* and 28 to *L. (V.) guyanensis*. However, the subsequent sequencing of 32 hsp70-PCR products (21 *L. (V.) panamensis* and 11 *L. (V.) guyanensis*) confirmed the typing of *L. (V.) panamensis*, but relocated the other 11 *L. (V.) guyanensis* sequences into a distinct group previously referred to as *Leishmania* sp.1 [17]. This limited capacity of PCR hsp70-RFLP to distinguish *L. (V.) guyanensis* from *Leishmania* sp.1 has already been reported [17]. These findings confirm that *L. (V.) panamensis* is the predominant species for this type of lesion, with a negative parasitic diagnosis. However, the results reveal that 40.6% of these cases belong to *Leishmania* sp.1 in an apparently higher proportion than was reported in a previous study (23.2%) [17]. So far, this genetic variant has only been described in Panamanian patients with LC, but its association with the clinical course, diagnosis, and epidemiological characteristics has not yet been evaluated [17]. According to our data, no significant differences were found between *L. (Viannia) panamensis* and *Leishmania* sp.1 when comparing the frequencies of demographic, epidemiological, and clinical variables which were analyzed in this study. Additionally, it was found that *Leishmania* sp.1 is also present in patients from regions where this species was not originally reported, such as the provinces of Colón and Panamá Oeste [17]. Thus, it is necessary to continue investigating the geographical distribution of *Leishmania* sp.1, its clinical features, induced immune response, and susceptibility to the pharmacological treatments used in Panama for CL.

One important limitation of this study was the relatively low number of samples evaluated, in addition to the fact that these DNA samples were remnants used for the routine diagnosis of suspected CL cases at the DIP-ICGES. For this reason, some of the samples that were initially characterized by PCR-Hsp70-RFLP yet possessed a low amount of an amplified product or extracted DNA could not proceed to the sequencing stage.

The results of this study reinforce the usefulness of molecular methods in the diagnosis and characterization of CL, especially in the case of lesions with a low parasitic load. In addition, this study identified the species/genetic variants of *Leishmania* involved, as well as some clinical/epidemiological characteristics linked to this type of lesion.

## Figures and Tables

**Table 1:** Evolution time and distribution of lesions by anatomical site.

Features	Total	Group A	Group B	X^2^	*p*-Value
Evolution time (Days)		N = 123	N = 123	5.514	0.1378
0–30	111 (45)	55 (45)	56 (46)		
31–60	72 (29)	34 (28)	38 (31)		
61–120	42 (17)	18 (15)	24 (20)		
>120 days	19 (8)	14 (11)	5 (4)		
Missing data	2 (1)	2 (2)	0 (0)		
FACE/NECK		N = 14	N = 21	0.972	0.3241
1 Lesion	30 (86)	11 (79)	19 (90)		
2 lesions	5 (14)	3 (21)	2 (10)		
BACK		N = 6	N = 12	2.819	0.2443
1 Lesion	13 (72)	5 (83)	8 (67)		
2 lesions	2 (11)	1 (17)	1 (8)		
>2 lesions	3 (17)	0 (0)	3 (25)		
ABDOMEN		N = 2	N = 7	1.148	0.284
1 Lesion	7 (78)	1 (50)	6 (86)		
2 lesions	2 (22)	1 (50)	1 (14)		
>2 lesions	0 (0)	0 (0)	0 (0)		
LEGS		N = 50	N = 30	5.41	0.1441
1 Lesion	61 (76)	42 (84)	19 (63)		
2 lesions	10 (13)	4 (8)	6 (20)		
3 to 5 lesions	8 (10)	4 (8)	4 (13)		
>5 lesions	1 (1)	0 (0)	1 (3)		
ARMS		N = 43	N = 69	4.84	0.1839
1 Lesion	84 (75)	34 (79)	50 (72)		
2 lesions	14 (13)	2 (5)	12 (17)		
3 to 5 lesions	11 (10)	5 (12)	6 (9)		
>5 lesions	3 (3)	2 (5)	1 (1)		

Data are No. (%) and were analyzed in JMP (SAS institute) software version 14, using Ji2 analyses. Significant differences (*p* ≤ 0.05).

## Data Availability

All data underlying results from this study are provided as part of the article in tables and figures.

## Supplementary Materials

The following supporting information can be downloaded at: https://www.mdpi.com/article/10.3390/tropicalmed7100282/s1, Table S1: Epidemiological data of the study cohort; Table S2: PCR -hsp70-RFLP data.
