# Supplementary material for: Characterization of Leishmania spp. Causing Cutaneous Lesions with a Negative Parasitological Diagnosis in Panama
Source: Trop Med Infect Dis. 2022 Oct 3;7(10):282. doi: 10.3390/tropicalmed7100282 (PMC9609048; doi:10.3390/tropicalmed7100282)
Supplement: Supplementary file 1 [file tropicalmed-07-00282-s001.zip › tropicalmed-1911091-supplementary.pdf]

Tropical Medicine and Infectious Disease

MDPI

# Supplementary Materials: Characterization of *Leishmania* spp. Causing Cutaneous Lesions with a Negative Parasitological Diagnosis in Panama

**Table S1.** Epidemiological data of the study cohort.

| Features | Total N = 246 (100%) | Group A N = 123 (100%) | Group B N = 123 (100%) | $X^2$ | *p-Value* |
|---|---|---|---|---|---|
| **Sex** | | | | | |
| M | 162 (66) | 80 (65) | 82 (67) | 0.072 | 0.788 |
| F | 84 (34) | 43 (35) | 41 (33) | | |
| **Age (Years)** | | | | | |
| 01–20 | 89 (36) | 47 (38) | 42 (34) | | |
| 21–50 | 115 (47) | 53 (43) | 62 (50) | 0.387 | 0.5 |
| 51–80 | 40 (16) | 22 (18) | 18 (15) | | |
| Missing data | 2 (1) | 1 (1) | 1 (1) | | |
| **Province** | | | | | |
| Panamá Oeste | 75 (30) | 34 (28) | 41(33) | | |
| Panamá | 107 (43) | 58 (47) | 49(40) | | |
| Colón | 41 (17) | 20 (16) | 21(17) | | |
| Darién | 3 (1) | 1 (1) | 2(1) | | |
| Bocas del Toro | 2 (1) | 1 (1) | 1(1) | 4.542 | 0.8052 |
| Chiriquí | 9 (4) | 5 (4) | 4(3) | | |
| Veraguas | 1 (0) | 0 (0) | 1(1) | | |
| Herrera | 1 (0) | 0 (0) | 1 (1) | | |
| Coclé | 6 (2) | 4 (3) | 2(2) | | |
| Missing data | 1 (0) | 0 (0) | 1(1) | | |
| Province of possible infection | | | | | |
| Los santos | 2 (1) | 0 (0) | 2 (2) | | |
| Colón | 50 (20) | 28 (23) | 22 (18) | | |
| Coclé | 20 (8) | 9 (7) | 11 (9) | | |
| Panamá Oeste | 72 (29) | 36 (29) | 36 (29) | | |
| Panamá | 50 (20) | 22 (18) | 28 (23) | | |
| Bocas del toro | 9 (4) | 7 (6) | 2 (2) | 7.903 | 0.6383 |
| Darién | 20 (8) | 11 (9) | 9 (7) | | |
| Veraguas | 3 (1) | 1 (1) | 2 (2) | | |
| Chiriquí | 3 (1) | 2 (2) | 1 (1) | | |
| Comarca Guna Yala | 3 (1) | 1 (1) | 2 (2) | | |
| Missing data | 14 (6) | 6 (5) | 8 (7) | | |

**Group A.** *Leishmania* with negative smears and/or culture; **Group B**. *Leishmania* with positive smears and/or culture. Data are No. (%) and were analyzed in JMP (SAS institute) software version 14, using $X^2$ analyses. Significant differences ($p$ = <0.05).

**Table S2.** PCR Hsp70-RFLP data.

| ***Leishmania* specie** | **Total N = 192 (100%)** | **Group A N = 69 (100%)** | **Group B N = 123 (100%)** | **$X^2$** | ***p-Value*** |
|---|---|---|---|---|---|
| *L. (V.) panamensis* | 140 (72.9) | 41 (59.4) | 99 (80.5) | 9.935 | **0.002** |
| *L. (V.) guyanensis* | 52 (27.1) | 28 (40.6) | 24 (19.5) | | |

**Group A.** *Leishmania* with negative smears and/or culture; **Group B**. *Leishmania* with positive smears and/or culture. Data are No. (%) and were analyzed in JMP (SAS institute) software version 14, using $X^2$ analyses. Significant differences ($p = <0.05$) are marked in bold.
